# [^18^F]FDG PET/MRI in children suffering from lymphoma: does MRI contrast media make a difference?

**DOI:** 10.1007/s00330-023-09840-5

**Published:** 2023-06-20

**Authors:** Kai Jannusch, Janna Morawitz, Bernd Schweiger, Daniel Weiss, Lars Schimmöller, Peter Minko, Ken Herrmann, Wolfgang P. Fendler, Harald H. Quick, Gerald Antoch, Lale Umutlu, Julian Kirchner, Nils-Martin Bruckmann

**Affiliations:** 1 Department of Diagnostic and Interventional Radiology, Medical Faculty, University Dusseldorf, Moorenstrasse 5, 40225 Dusseldorf, Germany; 2 Department of Diagnostic and Interventional Radiology and Neuroradiology, University Hospital Essen, University of Duisburg-Essen, 45147 Essen, Germany; 3 Department of Nuclear Medicine, University Hospital Essen, University of Duisburg-Essen, 45147 Essen, Germany; 4 High-Field and Hybrid MR Imaging, University Hospital Essen, University Duisburg-Essen, 45147 Essen, Germany; 5 Erwin L. Hahn Institute for Magnetic Resonance Imaging, University Duisburg-Essen, 45141 Essen, Germany

**Keywords:** Positron-emission tomography, Magnetic resonance imaging, Lymphoma, Neoplasm staging, Contrast media

## Abstract

**Objectives:**

Evaluate the influence of an MRI contrast agent application on primary and follow-up staging in pediatric patients with newly diagnosed lymphoma using [^18^F]FDG PET/MRI to avoid adverse effects and save time and costs during examination.

**Methods:**

A total of 105 [^18^F]FDG PET/MRI datasets were included for data evaluation. Two different reading protocols were analyzed by two experienced readers in consensus, including for PET/MRI-1 reading protocol unenhanced T2w and/or T1w imaging, diffusion-weighted imaging (DWI), and [^18^F]FDG PET imaging and for PET/MRI-2 reading protocol an additional T1w post contrast imaging. Patient-based and region-based evaluation according to the revised International Pediatric Non-Hodgkin’s Lymphoma (NHL) Staging System (IPNHLSS) was performed, and a modified standard of reference was applied comprising histopathology and previous and follow-up cross-sectional imaging. Differences in staging accuracy were assessed using the Wilcoxon and McNemar tests.

**Results:**

In patient-based analysis, PET/MRI-1 and PET/MRI-2 both determined a correct IPNHLSS tumor stage in 90/105 (86%) exams. Region-based analysis correctly identified 119/127 (94%) lymphoma-affected regions. Sensitivity, specificity, positive predictive value, negative predictive value, and diagnostic accuracy for PET/MRI-1 and PET/MRI-2 were 94%, 97%, 90%, 99%, 97%, respectively. There were no significant differences between PET/MRI-1 and PET/MRI-2.

**Conclusions:**

The use of MRI contrast agents in [^18^F]FDG PET/MRI examinations has no beneficial effect in primary and follow-up staging of pediatric lymphoma patients. Therefore, switching to a contrast agent–free [^18^F]FDG PET/MRI protocol should be considered in all pediatric lymphoma patients.

**Clinical relevance statement:**

This study gives a scientific baseline switching to a contrast agent–free [^18^F]FDG PET/MRI staging in pediatric lymphoma patients. This could avoid side effects of contrast agents and saves time and costs by a faster staging protocol for pediatric patients.

**Key Points:**

• *No additional diagnostic benefit of MRI contrast agents at [*^*18*^*F]FDG PET/MRI examinations of pediatric lymphoma primary and follow-up staging*

• *Highly accurate primary and follow-up staging of pediatric lymphoma patients at MRI contrast–free [*^*18*^*F]FDG PET/MRI*

## Introduction

As the third most common tumor disease, lymphomas represent a significant proportion of pediatric malignancies, accounting for approximately 15% [1]. Generally, lymphoma types are broadly distinguished: Non-Hodgkin’s lymphoma (NHL) is diagnosed more frequently than Hodgkin’s lymphoma (HL) [2]. Highly accurate staging of lymphoma patients plays an important role for therapy, as it is well known that therapeutic strategies and prognosis depend heavily on the tumor stage at initial staging [3–5]. Although contrast-enhanced computed tomography (CT) diagnostic is used frequently due to its immediate availability for initial staging, [^18^F]fluorodeoxyglucose-positron emission tomography ([^18^F]FDG PET)/CT is considered the current reference imaging method. The additional metabolic information outperforms single CT diagnostics especially for staging of small nodal lymphoma manifestations and for response assessment. Nonetheless, both modalities come with repetitive radiation exposure and an increasing risk of future secondary malignancies [6–9]. Especially for children, a radiation-saving diagnostic alternative should be the goal. Combining high spatial soft tissue resolution with a functional imaging dataset, [^18^F]FDG PET/magnet resonance imaging (MRI) is becoming increasingly important as a radiation-saving alternative for initial staging and follow-up imaging of all lymphoma patients, especially in children [10–14]. It enables a reduction of radiation exposure up to 65%, while obtaining high quality at morphologic lymphoma imaging [15–17]. Consequently, improvements of the diagnostic work-up therapeutic abilities build the basis of increasingly favorable prognosis for children with lymphoma, measurable with a 5-year survival of 97% for HL and 85% for NHL, respectively [18–20]. However, [^18^F]FDG PET/MRI can easily reach 1 h of examination time for an adequate staging, making it stressful and challenging for pediatric and adult patients [21]. Bearing in mind that lymphoma patients commonly undergo multiple examinations for staging and therapy monitoring, special attention should be paid to the ongoing discussion about the relevance of gadolinium deposition to the brain [15, 22–25]. Thus, application of an MRI contrast agent should be reduced to a clinically reasonable minimum. Thus, skipping contrast administration and the associated contrast-enhanced sequence could reduce the risk of adverse effects like the discussed gadolinium deposition in the brain and allergic reactions and save time as well as costs.

A pilot study published by Kirchner et al. in 2017 already compared different reading protocols in a small cohort, entailing non-enhanced/contrast-enhanced and diffusion-weighted [^18^F]FDG PET/MR imaging and whole-body diffusion-weighted MRI for lesion detection and determination of the tumor stage in pediatric lymphoma patients. This study revealed that the application of contrast agents does not lead to a noticeable improvement of the diagnostic accuracy of a PET/MRI staging [13]. Nonetheless, data about the value of MRI contrast agent application in [^18^F]FDG PET/MRI in the diagnostic work-up of pediatric lymphoma patient is still limited to few small cohort studies [26–29].

Therefore, the present follow-up study aims to further validate a potentially time- and contrast agent–saving [^18^F]FDG PET/MRI protocol in the diagnostic work-up of pediatric lymphoma patients.

## Material and methods

### Patients

The institutional review board (study number 11–4822-BO) approved this study and it was performed in conformance with the Declaration of Helsinki. All patients underwent a clinically indicated contrast-enhanced whole-body [^18^F]FDG PET/MRI after informed written consent of the parents was obtained. Histopathological verification of lymphoma subtypes was available in all patients. Following a publication of the Council on Child and Adolescent Health (1988) that sees the upper pediatric age limit at 21 and a 2019 published study of Arendt et al which included pediatric lymphoma patients until the age of 25 years, this study included pediatric lymphoma patients < 21 years [26, 30]. Ultimately *n* = 25 HL patients and *n* = 7 NHL patients mean aged 14 ± 3 years (range 7–20 years) with a total of 105 examinations, including scans for initial staging (*n* = 32) and restaging during treatment or at the end of treatment (*n* = 73), as recommended in the ESMO Guidelines, were included [31, 32].

### Whole-body PET/MRI

All [^18^F]FDG PET/MRI examinations were performed on an integrated 3-T PET/MRI system (Biograph mMR, Siemens Healthcare GmbH) with an average delay of 67 ± 19 min after [^18^F]FDG injection. To ensure blood glucose levels below 150 mg/dl, blood samples were obtained prior to injection of a bodyweight adapted dosage of [^18^F]FDG (4 MBq/kg bodyweight). Mean activity was 202 ± 53 MBq. Initial [^18^F]FDG PET/MRI staging was performed using a whole body protocol (including the head and limbs). All follow-up scan volumes generally covered the skull base to mid-thigh if the head and limbs were unsuspicious in initial staging.

PET data acquisition was performed in up to 5 bed positions by using an acquisition time of 4 min per bed position, depending on children height. PET images were reconstructed using the iterative ordered-subset expectation maximization (OSEM) algorithm (3 iterations, 21 subsets, Gaussian filter 4 mm, matrix size 344 × 344) [33]. Investigating possible differences between (PET/MRI-1) unenhanced PET/MRI (T2 weighted (w) imaging and/or T1w imaging, diffusion-weighted imaging (DWI)) and (PET/MRI-2) contrast-enhanced PET/MRI (T2w imaging and/or T1w pre-contrast imaging and T1w post-contrast imaging, DWI), the readers were asked to exclusively read the corresponding sequences in different combinations out of a longer protocol. Due to MRI protocol adjustments in clinical practice for time saving in some cases, a T2w sequence or a contrast-free T1w sequence was skipped at some pediatric patients. The MRI protocols were set up in accordance with clinical (age-dependent) standards, entailing different kinds of T1w and T2w sequences as well as a transversal DWI echo-planar imaging (EPI) (*b* values: 0, 500, and 1000 s/mm^2^). For contrast-enhanced imaging, a transverse volume interpolated breath-hold examination (VIBE) after intravenous administration of a gadolinium-based contrast medium (Dotarem; 0.05 mmol/kg bodyweight, literature accepted standard value) was acquired.

### Image analysis

Imaging datasets of PET/MRI-1 and PET/MRI-2 were evaluated using a dedicated OsiriX workstation (Pixmeo SARL). A board-certified radiologist and a board certified nuclear medicine physician with experience in hybrid (more than 5 years) and MR imaging (more than 5 years) performed reading. Imaging datasets of the [^18^F]FDG PET/MRI examination were analyzed in consensus. In general, reading was subdivided in two different reading sessions entailing the different datasets of PET/MRI-1 and PET/MRI-2. Each dataset was evaluated in a dedicated reading session in a random order with a minimum of 4 weeks apart to avoid recognition bias. Both readers were blinded to patient identity and results of initial or follow-up imaging. Readers were informed about pediatric lymphoma diagnosis and scan indication (initial staging or restaging).

First, readers should evaluate all typical areas for presence or absence of lymphoma manifestation. Lymph nodes were summarized to nodal groups comprising the head/neck (Waldeyer’s ring, bilateral cervical and bilateral supraclavicular), chest (bilateral infraclavicular, prevascular, aortopulmonary, paratracheal, pretracheal, subcarinal, posterior mediastinal, bilateral hilar, and retrocrural), axilla/extremities (bilateral axillary), abdomen (gastrohepatic, periportal, aortocaval, retrocrural, mesenteric, retroperitoneal, and paraaortic), and pelvis (bilateral common iliac, bilateral internal iliac, bilateral external iliac and bilateral inguinal). Lymphoma manifestations at the bilateral pleura, bilateral lung, bilateral breast, myocardium, liver, ovary, and bowel as well as bone lesions were classified as extranodal manifestation.

Afterwards, readers were asked to separately set a tumor stage for each dataset in accordance with the IPNHLSS [34].

No universally applied morphologic criteria for pediatric lymphoma manifestation have been established yet. In accordance with previous publications for lymphoma in adults and according to the Lugano classification, the following morphologic criteria for the manifestation of lymphoma were considered: nodal lesions with a nodal long-axis diameter greater than 1.5 cm (unidimensional measurement), cluster formation or mass-like lesions, and high signal intensity at DWI sequences on high *b* value (b = 1000 s/mm^2^) with correlating signal drop in the corresponding ADC map [35–37]. In addition, a homogeneously accentuated contrast enhancement of a lesion and the adjacent tissue was considered lymphoma suspicious. Due to the large number of possible lymphoma-affected regions, contrast enhancement was assessed visually and not determined by a cut-off.

The 5-point (Deauville) scale for interpretation of [^18^F]FDG PET and the revised staging and response criteria of the Lugano classification were entirely focused on adult lymphoma without reference to pediatric lymphoma entities [5, 38]. Nonetheless, for lesion characterization on [^18^F]PET, visually increased focal FDG uptake in comparison to background and mediastinum and higher than liver activity was considered indicative for involvement with active lymphoma in concordance with the 5-point scale of the Lugano classification.

### Standard of reference

All included patients suffered from a histologically proven lymphoma disease. Patients suffering from NHL and unclear bone marrow involvement underwent bone marrow biopsy according to the actual pediatric guidelines [39–41]. Due to clinical and ethical standards, a histological confirmation of each lymphoma-suspected lesion was not possible. Therefore, in a final consensus reading a modified standard of reference was established by the two experienced readers on a patient and region basis. Previous and follow-up cross-sectional imaging were provided for the final consensus reading, as it was already performed in previous publications, to enable accurate lesion characterization [13, 42, 43].

### Statistical analysis

SPSS Statistics 26 (IBM Inc.) was used for statistical analysis. Data analysis was performed patient-based and region-based. Descriptive analysis was performed and data are presented as mean ± SD. Sensitivity, specificity, positive predictive value, negative predictive value, and diagnostic accuracy were calculated for PET/MRI-1 and PET/MRI-2, respectively. Confidence intervals were calculated at 95%. The Wilcoxon test was chosen for evaluation of differences in tumor stage between PET/MRI-1 and PET/MRI-2. The McNemar test was used for paired-group comparison at a region-based analysis. *P* values < 0.05 were considered to be statistically significant.

## Results

In total, *n* = 32 patients underwent [^18^F]FDG PET/MRI providing a total of 105 examinations. Mean acquisition time of contrast-enhanced PET/MRI examinations was 51 ± 23 min (range: 22 to 112 min) with a mean acquisition time of 2 ± 1 min (range: 1 to 6 min) of the T1-weighted post-contrast sequence. In all 105 examinations, MRI contrast–enhanced MRI was successfully completed. Eighty of total 105 (76%) examinations suffered from Hodgkin lymphoma, 4/105 (4%) examinations suffered from Burkitt lymphoma, 11/105 (10%) examinations suffered from B cell lymphoma, and 10/105 (10%) examinations suffered from T cell lymphoma (see Table 1).

**Table 1:** *N* = 105 pediatric lymphoma examinations subdivided in histological subtypes

Histological subtypes		Number of examinations
Hodgkin lymphoma		80/105 (76%)
Non-Hodgkin lymphoma	Burkitt lymphoma	4/105 (4%)
	B-cell lymphoma	11/105 (10%)
	T-cell lymphoma	10/105 (10%)

### Region-based analysis

As previously described, PET/MRI-1 and PET/MRI-2 imaging datasets of pediatric lymphoma patients were subdivided into six anatomical regions to differentiate between different anatomical lymphoma manifestations. According to the standard of reference, active lymphoma manifestations were visible at 127 anatomical regions. PET/MRI-1 and PET/MRI-2 correctly detected 119/127 (94%) lymphoma-affected regions. A detailed evaluation of active lymphoma manifestations according to the six anatomical regions is shown in Table 2.

**Table 2:** Correctly detected lymphoma-affected regions of all pediatric patients at PET/MRI-1 and PET/MRI-2 reading protocols according to the standard of reference

Lymphoma-affected regions	Standard of reference *n*	PET/MRI-1 *n*—correct	PET/MRI-2 *n*—correct
Head and Neck	42/127 (33%)	41/127 (32%)	41/127 (32%)
Chest	37/127 (29%)	35/127 (28%)	35/127 (28%)
Axilla and Extremities	13/127 (10%)	13/127 (10%)	13/127 (10%)
Abdomen	12/127 (9%)	10/127 (8%)	10/127 (8%)
Pelvis	4/127 (3%)	4/127 (3%)	4/127 (3%)
Extranodal	19/127 (15%)	16/127 (13%)	16/127 (13%)
Total	127	119/127 (94%)	119/127 (94%)

Sensitivity, specificity, positive predictive value, negative predictive value, and diagnostic accuracy for PET/MRI-1 and PET/MRI-2 were 94%, 97%, 90%, 99%, and 97%, respectively (see Table 3). No statistically significant difference was seen between both reading protocols (*p* = 1).

**Table 3:** Quality criteria of PET/MRI-1 and PET/MRI-2 subdivided in sensitivity, specificity, positive predictive value, negative predictive value, and diagnostic accuracy

	PET/MRI-1 (%)	PET/MRI-2 (%)
Sensitivity	94 (CI 89–98)	94 (CI 89–98)
Specificity	97 (CI 95–98)	97 (CI 95–98)
Positive Predictive Value	90 (CI 83–94)	90 (CI 83–94)
Negative Predictive Value	99 (CI 97–99)	99 (CI 97–99)
Accuracy	97 (CI 95–98)	97 (CI 95–98)

### Patient-based analysis

**Fig. 1:**
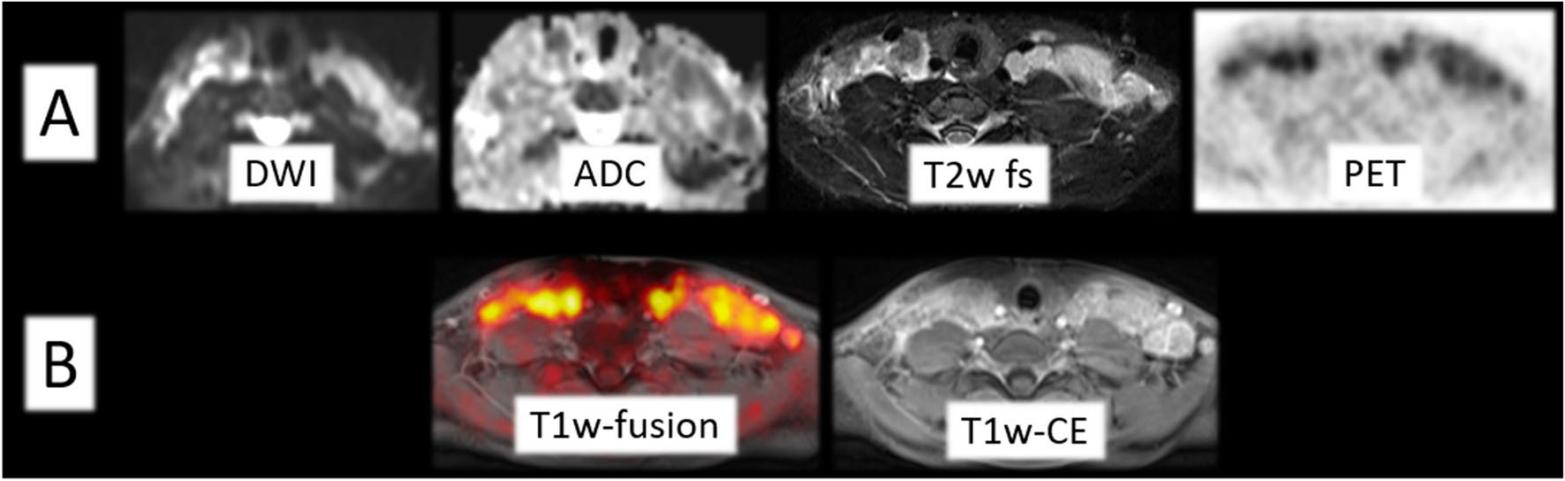
Example of a 15-year-old pediatric patient with correctly identified active lymphoma disease at PET/MRI-1 (**A**) and PET/MRI-2 (**A**, **B**) with visual [^18^F]FDG uptake (SUVmax: 13.1). No additional diagnostic benefit of the T1-weighted (w) contrast-enhanced (CE) sequence was seen

**Fig. 2:**
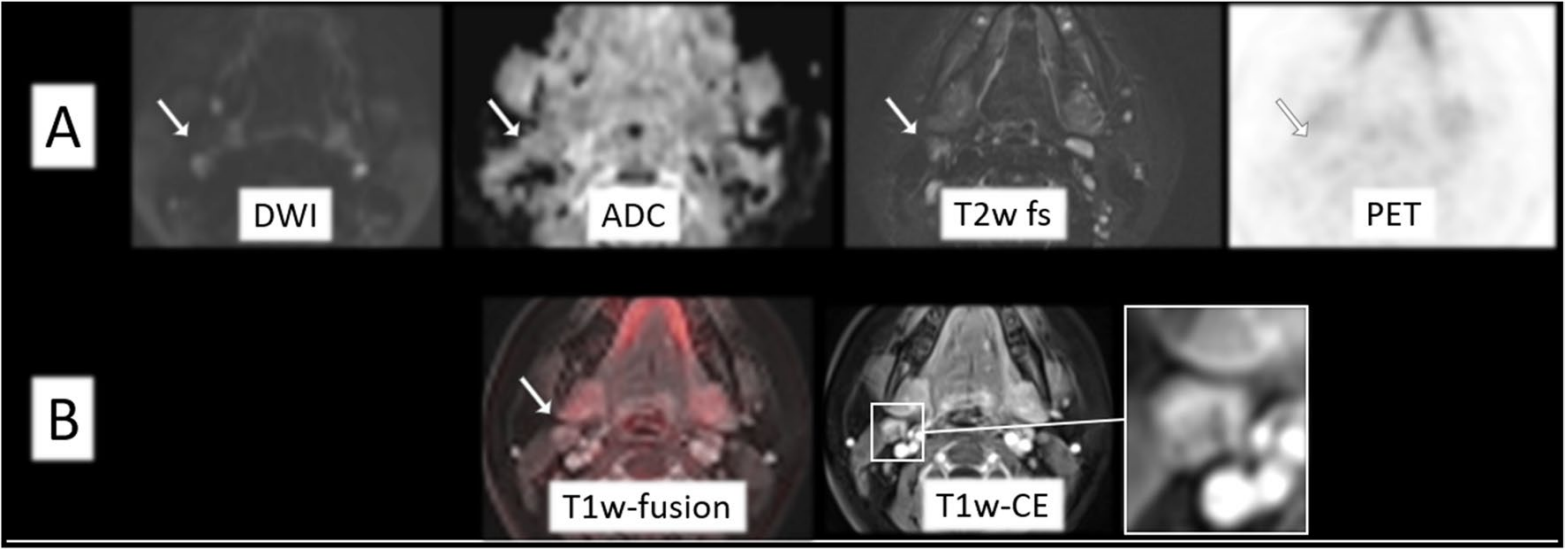
Example of a 13-year-old pediatric patient with missed active lymphoma disease at PET/MRI-1 (**A**) and PET/MRI-2 (**A**, **B**) according to the standard of reference. No visual [^18^F]FDG uptake (SUVmax: 2.0) was seen. Non-pathological appearance of the lymph node right cervical with borderline size (13 mm). Minimal contrast agent accumulation (white frame) within the lymph node in the T1-weighted (w) contrast-enhanced (CE) sequence. However, in synopsis of the images, this did not change the evaluation

According to the standard of reference, active lymphoma was present in 65/105 (62%) examinations and 40/105 (38%) examinations had no evidence of disease (see Table 4). Sixty-two of 65 active lymphoma manifestations were identified by PET/MRI-1 and PET/MRI-2, and the same three were missed respectively (exemplified in Figs. 1 and 2). PET/MRI-1 and PET/MRI-2 each rated the same three examinations as false positive (3/40; 8%).

**Table 4:** Presentation of IPNHLSS tumor stages determined by standard of reference, correctly identified at PET/MRI-1 and PET/MRI-2 examinations and over-/underrated at PET/MRI-1 and PET/MRI-2 according to the standard of reference

IPNHLSS tumor stage	Standard of reference *n*	PET/MRI-1 *n*—correct	PET/MRI-2 *n*—correct
No evidence of disease	40/105 (38%)	36/105 (34%)	36/105 (34%)
1	5/105 (5%)	3/105 (3%)	3/105 (3%)
2	16/105 (15%)	15/105 (14%)	15/105 (14%)
3	34/105 (32%)	30/105 (29%)	30/105 (29%)
4	10/105 (10%)	6/105 (6%)	6/105 (6%)
*n*—overrated		7/105 (7%)	7/105 (7%)
*n*—underrated		8/105 (8%)	8/105 (8%)

Patient-based lymphoma manifestation was determined in accordance with the revised IPNHLSS. According to the standard of reference, 40/105 (38%) examinations had no evidence of disease, 5/105 (5%) examinations had an IPNHLSS stage 1, 16/105 (15%) examinations had an IPNHLSS stage 2, 34/105 (32%) examinations had an IPNHLSS stage 3, and 10/105 (10%) examinations had an IPNHLSS stage 4 lymphoma manifestation. PET/MRI-1 and PET/MRI-2 determined a correct IPNHLSS tumor stage in 90/105 (86%) examinations and each protocol overrated 7 of 105 (7%) lymphoma-affected examinations. The same 8 examinations of total 105 (8%) were underrated in both reading protocols. There was no significant difference determining IPNHLSS stage between both reading protocols (*p* = 1). The rated lymphoma stages of the PET/MRI-1 and PET/MRI-2 are given in Table 4.

## Discussion

[^18^F]FDG PET/MRI is increasingly accepted for staging of lymphoma patients, and the present study further confirms the feasibility and high diagnostic accuracy of it in pediatric lymphoma patients [11, 12]. Especially in larger lymphoma treatment centers, [^18^F]FDG PET/MRI becomes more and more the diagnostic of choice in the work-up of lymphoma patients compared to the current [^18^F]FDG PET/CT reference imaging method. This is also caused by the fact that radiation exposure when using [^18^F]FDG PET/MRI is significantly less than the radiation exposure of even a low-dose [^18^F]FDG PET/CT [44, 45]. Bearing in mind the repetitive scans for therapy planning and follow-up imaging a lymphoma patient needs to undergo the reduction in radiation exposure using [^18^F]FDG PET/MRI is even more substantial. Consequently, the risk of secondary malignancies due to radiation exposure becomes significantly lower [6, 7]. This is of particular high importance in pediatric patients.

The need for MRI contrast agent application in PET/MRI is clinically not debatable in individual tumor entities for further classification, such as liver or pelvic tumors [46–48]. Contrary, first publications have already shown that especially in pediatric lymphoma patients, a MRI contrast agent application might be waived for staging [13, 29]. MRI contrast–free [^18^F]FDG PET/MRI imaging protocols are able to outperform a radiation-saving [^18^F]FDG PET/CT low-dose protocol with a more accurate soft tissue contrast and its well-known advantages when imaging parenchymal organs [13, 29]. Furthermore, they profit from simultaneously acquired metabolic PET imaging data that has been shown to be beneficial for staging, therapy monitoring, and differentiation between active and non-active lymphoma diseases [15–17, 49].

The omission of contrast administration and the associated contrast-enhanced sequence would avoid side effects of contrast agents like gadolinium deposition to the body, allergic reactions, and incompatibilities and furthermore saves time and costs by a faster [^18^F]FDG PET/MRI protocol [24]. Thus, the present follow-up study aims to further validate the need of MRI contrast agent application in the diagnostic [^18^F]FDG PET/MRI work-up of pediatric lymphoma patients by evaluating two different reading protocols.

According to our data evaluation, there was no difference between the diagnostic potential of PET/MRI-1 and PET/MRI-2. Bearing in mind that this cohort consists of initial and follow-up examinations, it can be concluded that pediatric lymphoma patients do not benefit from MRI contrast agent application at initial and follow-up [^18^F]FDG PET/MRI staging. In detail, information about vascularization gained from contrast MRI sequences does not add information when compared to FDG metabolic identification, MRI diffusion, and morphological localization of lymphoma. Clinical relevant aspects of lymphoma staging for therapy planning are metabolic mapping and morphologic information on size, shape, and infiltration we can achieve from non-contrast [^18^F]FDG PET/MRI without missing therapeutic relevant information. Apart from tumor detection, the application of contrast agents might be beneficial in cases of large mediastinal tumors, e.g., causing a superior vena cava compression syndrome. A venous vessel compression can lead to venous thrombosis that are better delineated after contrast agent application [50, 51]. However, since these cases are very rare, administration of contrast agents should be strictly discussed and regulated in the future due to the lack of diagnostic benefit shown in this study. For example, administration after clinical examination and suspicion of venous occlusion may be appropriate accordingly.

Figure 2 visualizes one borderline patient with missed lymphoma manifestation cervical (right) at PET/MRI-1 and PET/MRI-2 according to the standard of reference. Although MRI contrast agent uptake might result in a better delineation and potentially give a hint to subsequent lymphoma manifestation, there is no clinical benefit in this case. Given the morph on both reading protocols and without visible [^18^F]FDG uptake, this lymph node would not be considered suspicious for lymphoma in a clinical setting.

Dependent on the aggressiveness of lymphoma disease, sensitivity and accuracy of whole-body MRI and [^18^F]FDG PET/CT in detecting bone marrow involvement of adult and pediatric patients is ranging from 45 to 100% [52–55]. Generally, focal or multifocal [^18^F]FDG uptake exceeding liver uptake indicates bone marrow involvement of lymphoma in adults [56]. Diffuse [^18^F]FDG uptake of the bone marrow is more likely associated with lymphoma bone marrow manifestation in NHL than in HL [56, 57]. In our data, it is noticeable that a lower number of lymphomas were detected in examinations who suffered from a stage 4 IPNHLSS lymphoma (bone marrow involvement, 6/105 vs. 10/105) compared to the standard of reference including a bone marrow aspiration for unclear involvement and NHL patients. Missed children with bone marrow involvement of their lymphoma showed a more diffuse [^18^F]FDG uptake of the bone marrow in our data. In addition to a general misinterpretation by the reader during the data evaluation, another possible explanation for this underestimation of bone marrow involvement could be the increased proportion of the [^18^F]FDG affine red bone marrow in children compared to adults [58]. During adolescence, the proportion of yellow bone marrow increases, resulting in a decreased metabolic FDG activity of the bone. However, this may also increase again in adults, especially when suffering from hematopoietic diseases. Thus, yellow bone marrow is again converted to red bone marrow for hematopoiesis at patients with anemia, especially common at HL [57]. Furthermore, diffuse inflammatory bone marrow reaction also visualized at lymphoma patients makes it also difficult to identify bone marrow involvement of lymphoma [57]. Although the partially different, anatomical condition supports the fact that staging of adult lymphoma patients cannot be unreservedly transferred to staging of pediatric lymphoma patients, subtle bone marrow involvement of pediatric patients could be more difficult to detect at diffuse [^18^F]FDG uptake of the bone marrow exceeding liver uptake according to the described findings [13].

In line with the current literature, both reading protocols had a high sensitivity, specificity, positive predictive value, negative predictive value, and accuracy above 89% concordant to previous publications [36, 59]. Our results clearly support the increasing trend of MRI contrast agent–free [^18^F]FDG PET/MR imaging in pediatric lymphoma patients [26–29]. This is from particular importance to skip the potential risk of gadolinium deposition to in the brain (dentate nucleus/paleostriatum) and bone due to repetitive application of linear, gadolinium-based contrast agents [23, 24, 60]. Although there is no evidence to date concerning adverse effects of gadolinium-based contrast agents or clinical implications, the potential risk makes it necessary to reduce the MRI contrast agent to a justifiable minimum [24].

In addition, [^18^F]FDG PET/MRI is a time-consuming examination that can easily reach 1 h of examination time as visualized at our acquisition times (mean: 51 min) and can be hard to challenge, especially for children [29]. There is a need of a compromise between short examination times and adequate image quality. Especially in children the duration of a PET/MRI examination should be as short as possible to increase acceptance and decrease potential anesthesia [61]. On the one hand shortened PET image acquisition times and on the other hand the adaption of the MRI protocol can manage this problem by ending up with a “fast”-PET/MRI protocol [36, 62, 63]. Our data highly support adjusting the MRI part by omitting the post-contrast whole-body T1-weighted MRI sequence for staging. This would save up to 6 min according to the evaluated data. Whole-body DWI might be beneficial for staging, as it seems to be a promising, radiation-free staging alternative of lymphoma patients with nearly same diagnostic abilities compared to PET/CT examinations [27, 64, 65]. Nonetheless, not all study results support the use of whole-body DWI for pediatric lymphoma staging. Thus, a study of Shapira-Zaltsberg and colleagues with focus on pediatric HL highlights the superiority of PET/CT at initial staging and assessment of therapy response [66]. However, at comprehensive [^18^F]FDG PET/MRI staging of pediatric lymphoma patients, whole-body DWI seems to have no beneficial effect for staging [13]. This might be due to the included PET component that is highly accurate in the detection of lymphoma manifestations. Furthermore, the results by Georgi et al. recommend the high diagnostic potential of T2-weighted transverse fat-saturated sequence for sufficient staging of pediatric lymphoma patients [29]. Additionally, a potential reduction of the PET acquisition times to 2 min is possible as described by Hartung-Knemeyer et al. [67]. Taking all this information into account, a relevant reduction of [^18^F]FDG PET/MRI examination times could be achieved.

[^18^F]FDG PET/MRI is predestined for children suffering from lymphoma and should be considered the diagnostic of choice. Since these patients are often treated in larger centers for pediatric medicine, these data will help to establish PET/MRI diagnostics at these. Furthermore, the feasibility of adapting the [^18^F]FDG PET/MRI examination protocol, which is highlighted by the available publications, may improve patient satisfaction and reduce potential anesthesia, bearing in mind that the patients are children suffering from a severe disease. In this context, higher costs and/or financial aspects should rather be considered secondary and the aim of such examination protocols should be a potential risk reduction to a minimum.

This study is not without limitations. Although histopathological sampling for subtype determination was available in all patients, in accordance with current ethical and clinical guidelines, not every detected lesion could be sampled. Hence, as already published in numerous previous studies on hybrid imaging, a modified standard of reference was applied. Secondly, lymphoma is known to comprise a heterogeneous group of cancers entailing different subtypes. The limited number of pediatric lymphoma patients does not enable a further subgroup comparison because of underpowered statistical analyses. Furthermore, due to the diagnostic focus on HL patients at PET/MRI of the institute, retrospectively HL were predominantly included for data evaluation. Moreover, due to missing QOL data based on the retrospective design, impressions of children undergoing PET/MRI could not be implemented in data evaluation. Nonetheless, this study is one of the larger data collections related to pediatric lymphoma patients at a single institute.

Finally, the use of MRI contrast agents in [^18^F]FDG PET/MRI examinations does not add relevant diagnostic information in primary and follow-up staging of pediatric lymphoma patients. Therefore, switching to a contrast agent–free [^18^F]FDG PET/MRI protocol should be considered in all pediatric lymphoma patients.

## Abbreviations

HL: Hodgkin’s lymphoma
IPNHLSS: International Pediatric Non-Hodgkin’s Lymphoma Staging System
NHL: Non-Hodgkin’s lymphoma

## Declarations

### Guarantor

The scientific guarantor of this publication is Julian Kirchner.

### Statistics and biometry

One of the authors has significant statistical expertise.

### Study subjects or cohorts overlap

No study subjects or cohorts have been previously reported.

### Methodology

• Retrospective data evaluation
